# Deep brain stimulation of globus pallidus internus and subthalamic nucleus in Parkinson’s disease: a multicenter, retrospective study of efficacy and safety

**DOI:** 10.1007/s10072-023-06999-z

**Published:** 2023-08-09

**Authors:** Michele Mainardi, Dario Ciprietti, Manuela Pilleri, Giulia Bonato, Luca Weis, Valeria Cianci, Roberta Biundo, Florinda Ferreri, Massimo Piacentino, Andrea Landi, Andrea Guerra, Angelo Antonini

**Affiliations:** 1 Parkinson and Movement Disorders Unit, Study Center for Neurodegeneration (CESNE), Department of Neuroscience, University of Padua, Via Giustiniani 2, 35128 Padua, Italy; 2 Service of Neurology, Villa Margherita-Santo Stefano Private Hospital, Arcugnano, Italy; 3 Department of General Psychology, University of Padua, Via Giustiniani 2, 35128 Padua, Italy; 4 Unit of Neurology, Unit of Clinical Neurophysiology, Department of Neuroscience, University of Padova, 35128 Padova, Italy; 5 Department of Neurosurgery, AULSS 8 Berica Ospedale San Bortolo, Viale Rodolfi, 37 36100 Vicenza, Italy; 6 Academic Neurosurgery, Department of Neurosciences, University of Padova, 35128 Padova, Italy

**Keywords:** Deep brain stimulation, Parkinson’s disease, Motor complications, STN, GPi

## Abstract

**Background:**

Deep brain stimulation (DBS) is an established therapeutic option in advanced Parkinson’s disease (PD). Literature data and recent guidelines remain inconclusive about the best choice as a target between the subthalamic nucleus (STN) and the globus pallidus internus (GPi).

**Materials and methods:**

We retrospectively reviewed the clinical efficacy outcomes of 48 DBS-implanted patients (33 STN-DBS and 15 GPi-DBS) at a short- (<1 year from the surgery) and long-term (2–5 years) follow-up. Also, clinical safety outcomes, including postoperative surgical complications and severe side effects, were collected.

**Results:**

We found no difference between STN-DBS and GPi-DBS in improving motor symptoms at short-term evaluation. However, STN-DBS achieved a more prominent reduction in oral therapy (l-dopa equivalent daily dose, *P* = .02). By contrast, GPi-DBS was superior in ameliorating motor fluctuations and dyskinesia (MDS-UPDRS IV, *P* < .001) as well as motor experiences of daily living (MDS-UPDRS II, *P* = .03). The greater efficacy of GPi-DBS on motor fluctuations and experiences of daily living was also present at the long-term follow-up. We observed five serious adverse events, including two suicides, all among STN-DBS patients.

**Conclusion:**

Both STN-DBS and GPi-DBS are effective in improving motor symptoms severity and complications, but GPi-DBS has a greater impact on motor fluctuations and motor experiences of daily living. These results suggest that the two targets should be considered equivalent in motor efficacy, with GPi-DBS as a valuable option in patients with prominent motor complications. The occurrence of suicides in STN-treated patients claims further attention in target selection.

## Introduction

Deep brain stimulation (DBS) is an effective therapeutic option for advanced Parkinson’s disease (PD) [1, 2] but the choice between the two most commonly selected target nuclei, globus pallidus internus (GPi) and subthalamic nucleus (STN), is still debated [3, 4]. The recent EAN/MDS-ES guidelines on invasive PD treatment conclude that both STN- and GPi-DBS are effective to treat symptoms of advanced PD with fluctuations, but dopaminergic medications can be reduced more with STN-DBS [3]. However, the general perception in many specialized institutions, especially in Europe, is that GPi currently represents the target of choice in older patients, mainly because the literature suggests a lower incidence of neuropsychiatric complications compared to STN-DBS. Conversely, patients with a younger age at onset are often implanted in STN, because of its significant efficacy in lowering total l-dopa dose and thus reducing the occurrence of motor-response fluctuations [4–6].

The presence of dyskinesia represents an important feature in the choice of the target. In fact, STN-DBS-induced dyskinesia have been described in several patients and are linked to the efficacy of stimulation, thus being similar to drug-induced dyskinesia. Although involuntary movements are reversible when the stimulation is stopped, in some patients a paradoxical situation arises, called “brittle dyskinesia,” in which the choice of parameters is bounded to sub-optimal options to avoid the onset of the dyskinetic symptoms [7]. On the contrary, GPi-DBS is believed to have a better intrinsic anti-dyskinetic effect, probably due to specific physiological mechanisms of the targeted structure [8]. Another important factor in target selection is a proper pre-operatory neuropsychological assessment of the patient. STN-DBS has been associated with a higher incidence of cognitive adverse effects (particularly in the language domain) compared to GPi, which could make the latter option preferable in patients with pre-existing cognitive and/or psychiatric comorbidities or susceptibility [9]. Lastly, the effect of DBS on genetic PD is still debated, and it could become a future selection factor for surgery [10].

Recent European/Movement Disorders Group guidelines for advanced PD treatment suggested no difference between the effect of GPi stimulation and STN stimulation, except for drug reduction [11].

Our study aims to evaluate the clinical efficacy of DBS and compare the outcome between patients treated with GPi-DBS and STN-DBS, both in the short term (within 1 year from implantation) and, for a subset of patients, also in the long term (between 2 and 5 years). We paid particular attention to motor symptoms (measured by MDS-UPDRS III) and l-dopa-related complications (measured by MDS-UPDRS IV). Concerning clinical safety outcomes, we considered the rate of postoperative surgical complications (such as infections, brain lesions, ischemic strokes, or hemorrhages) and severe side effects related to stimulation including suicide or onset of psychiatric disorders.

## Materials and methods

### DBS cohort at baseline

In our study, we included 48 patients, who underwent DBS from 2016 to 2021, from two specialized advanced Parkinson referral centers in the Veneto region (University Hospital of Padova and Hospital of Vicenza). For each participant, the following demographic and clinical data were collected at baseline: gender, age at PD diagnosis, age and disease duration at the time of surgery, motor and disability rating scales assessed by the Movement disorders society-sponsored revision of the unified Parkinson’s disease rating scale (MDS-UPDRS) (both ON-medication ad OFF-medication), stage of functional disability assessed by Hoehn–Yahr scale, and daily dopaminergic drug dose (calculated with l-dopa equivalent daily dose-LEDD) [12, 13]. The two centers, as indicated in the CAPSIT protocol [2], performed a complete neuropsychological assessment evaluating executive functions, memory, language, and functional disability (using activities daily living-ADL) to exclude the presence of dementia [14, 15]. Patients with early onset (<45 years of old) and/or family history of dementia or Parkinson’s disease were proposed to undergo genetic testing with a next generation sequencing (NGS) panel including the following genes: CHCHD2, DNAJC6, FBXO7, GBA, LRRK2, PARK2, PARK7, PINK1, SNCA e SYNJ1, ATP13a2, VPS13, NPC2. Twenty-eight patients out of 48 underwent the test.

### Surgery and programming

All the patients underwent neurosurgery for DBS either at the University Hospital of Padova or at the Hospital of Vicenza. On the first day after surgery, every patient underwent clinical evaluation for quantification of stun-effect and oral therapy adjustment: in STN-DBS patients a generally more significant stun-effect was detected, which led to an empirical average reduction of oral therapy of approximately 30% LEDD, while for GPi-DBS patients the oral dopaminergic therapy remained relatively unchanged in the immediate post-surgical phase. After 2–3 weeks from surgery, all the patients underwent monopolar review for optimal contact selection, based on clinical response and absence of stimulation-induced adverse effects. No major differences between the two targets were found during initial programming at the time of onset and general efficacy on motor symptoms.

### Follow up for efficacy and safety measures

We divided the follow-up period into two different assessments: a short-term evaluation (within 1 year from surgery) and, for a subset of patients (11 with STN-DNS and 10 with GPi-DBS), a long-term evaluation (2 to 5 years after surgery). For each subject, we collected the following efficacy measures: (1) changes in MDS-UPDRS subtotal scores (part I, non-motor aspects of experiences of daily living; part II, motor aspects of experiences of daily living; part III, clinical motor examination; part IV, historical and objective motor complication); (2) changes in functional disability (as measured by the Hoehn–Yahr scale); (3) changes in LEDD. Absolute variations from baseline to follow-up at each time point were calculated. Regarding side effects, we considered the frequencies of (1) postoperative surgical complications such as infections, brain lesions, ischemic strokes, or hemorrhages; (2) the onset of severe side effects due to stimulation, such as suicides or other psychiatric disorders (e.g., delusions, onset of impulse control disorder). We did not collect mild side effects related to the stimulation (such as transient sensory problems or speech disorders).

### Statistical methods

Baseline demographic characteristics were compared between the two groups (GPi-DBS vs. STN-DBS) using the *Mann-Whitney U Test* and *Fisher Exact Test*, whenever appropriate. Short-term vs. baseline and long-term vs. baseline clinical efficacy measures were analyzed using the *Wilcoxon Test*. To search for statistically significant differences in the magnitude of absolute variations, we calculated the difference between the value at follow-up and baseline for each endpoint item in each patient. Then, we compared the distributions of absolute variations between the two populations at each time point using the *Mann-Whitney U Test.* A two-tailed level of *P* ≤ .05 was considered significant in the statistical analysis. The results were summarized using descriptive statistics. Quantitative data were expressed as median (inter quartile range, IQR).

## Results

### Baseline evaluation

Our population included 48 patients (28 males and 20 females), 33 treated with STN-DBS, and 15 with GPi-DBS. All patients were referred to DBS surgery for long-term l-dopa therapy complications (motor fluctuations and dyskinesia), according to CAPSIT protocol [2]. In particular, patients were given an indication for GPi-DBS if they presented with a higher degree of severity for dyskinesia. The baseline characteristics of the two groups were for the most part similar, as reported in Table 1. Significant differences at baseline were found for MDS-UPDRS II (STN-DBS median value: 8, IQR: 5 vs. GPi-DBS median value 12, IQR: 5.5) and MDS-UPDRS IV scores (STN-DBS median value: 11, IQR: 8 vs. GPi-DBS median value 15, IQR: 10). This difference probably reflected the aforementioned pre-operative selection criteria in the two groups. Also, there was a higher proportion of female patients in the GPi-DBS than the STN-DBS group (*P* < 0.05), probably due to a higher prevalence of dyskinesia in the former population [16].

**Table 1:** Differences between STN and GPi groups at baseline evaluation

	STN-DBS (*N* = 33) Median (IQR)	GPI-DBS (*N* = 15) Median (IQR)	*P* value
Sex (M%)	72.7%	26.6%	***P*** **< .05**
Age of onset (years)	47 (12.5)	48 (9.5)	*P* = .30
Years of disease at surgery (years)	12 (5)	9 (3.5)	*P* = .11
Age at surgery (years)	58 (14)	61 (11.5)	*P* = .60
Surgery ≤ 10 yrs from onset (%)	42%	73.3%	*P* = .06
MDS-UPDRS I	8 (5)	12 (5.5)	*P* = .06
MDS-UPDRS II	11 (8)	15 (10)	***P*** **= .04**
MDS-UPDRS III ON-MED	18 (12)	17 (19.5)	*P* = .79
MDS-UPDRS III OFF-MED	34 (16)	38 (19)	*P* = .83
MDS-UPDRS IV	8 (4)	14 (5)	***P*** **= .001**
H-Y Scale	3 (1)	3 (1)	*P* = .86
LEDD	1000 (550.5)	1000 (247.125)	*P* = .57
ADL	6 (1)	6 (1)	*P* = .76
MMSE (corrected score)	26.2 (1.8)	26.2 (2.55)	*P* = .99

Significant differences at *P* < .05 are reported in bold

Twenty-eight (2 patients with GPi-DBS and 26 STN-DBS) out of 48 patients underwent genetic testing with an NGS panel. These patients had young onset PD (≤45 years) or positive family history of PD or dementia. Eleven patients (39.2%) had at least one variant in known PD-associated genes (Table 2), and 8 of them had a definite diagnosis. Pathogenic heterozygous *GBA* mutations were found in 4 patients, and one subject had a pathogenic *LRRK2* mutation; as for recessive genes, one patient had a homozygous mutation of *Park2* and two had single variants of the same gene (possible risk factor). One patient had already been tested and reported by another center using whole exome sequencing resulting in mutations in *PINK1* [18]. A single variant of uncertain significance was found in the *NPC2* gene.

**Table 2:** Patients with variants in PD-associated genes. Demographic characteristics, age at onset, disease duration, DBS target, involved gene, mutation, and its effect according to new American College of Medical Genetics (ACMG) classification [17] are shown. VUS: variant of unknown significance

N° patient, sex	Age at onset	Disease duration	Gene	Type of mutation	Effect	ACMG classification	DBS target
1, M	37	18	GBA	c.1226A>G p.Asn409Ser (N370S variant)	Missense	Pathogenic	STN
2, M	26	15	GBA	c.115+1G>A	Splicing	Pathogenic	STN
3, M	47	11	NPC2	c.58G>T p.(Glu20*)	Truncating	VUS	STN
4, M	43	15	Park2	c.1204C>T p.Arg402Cys	Missense	Risk factor	GPi
5, M	19	29	Park2	c.535_618del p.(Gly179_Ala206del)	Deletion	Pathogenic	STN
6, M	44	13	GBA	c.413delC p.(Pro138Leufs*62)	Truncating	Pathogenic	STN
7, F	39	22	PINK1	g.15445_15467del23	Deletion	Pathogenic	STN
8, F	43	9	GBA +SNCA	GBA (p.Asn409Ser N370S); SNCA (p.Ala17Asp)	Missense	GBA pathogenic, SNCA VUS	GPi
9, M	47	8	LRRK2	c.5606T>C p.(Met1869Thr)	missense	Pathogenic	STN
10, M	47	9	GBA	c.1226A>G p.(Asn409Ser). N370S variant	Missense	Pathogenic	STN
11, M	39	7	Park2	c.823C>T p.Arg275Trp	Missense	Risk factor	STN

### Short-term efficacy evaluation

Forty-eight patients were evaluated at short term, including 33 patients who underwent STN-DBS and 15 who underwent GPi-DBS. Median (IQR) scores at clinical assessment in the whole cohort are summarized in Table 3. Patients who underwent STN-DBS experienced a significant improvement in motor symptoms severity (MDS-UPDRS III ON: median 18, IQR 12 vs. median 12, IQR 12, *P* = .009; MDS-UPDRS III OFF: median 34, IQR 16 vs. median 21, IQR 16.5, *P* = .0003), in motor complications (MDS-UPDRS IV: median 8, IQR 4 vs. median 6, IQR 5, *P* = .0008), and a marked reduction in total LEDD (median 1000, IQR 550.5 vs. median 656, IQR 629, *P* = .0001). There was no statistically significant difference in MDS-UPDRS I, MDS-UPDRS II score, or H-Y stage from baseline.

**Table 3:** For each target, the table reports median value for each endpoint item at baseline and short-term evaluation

	STN			GPi		
	Pre DBS Median (IQR)	Short term Median (IQR)	*P* value	Pre DBS Median (IQR)	Short term Median (IQR)	*P* value
MDS-UPDRS I	8 (5)	7 (5)	*P* = .18	12 (5.5)	9 (10)	*P* = .47
MDS-UPDRS II	11 (8)	10 (5)	*P* = .15	15 (10)	10 (8.5)	***P*** **< .01**
MDS-UPDRS III ON	18 (12)	16 (12)	***P*** **< .01**	17 (19.5)	12 (14)	*P* = .07
MDS-UPDRS III OFF	34 (16)	21 (16.5)	***P*** **< .001**	39 (20.5)	25.5 (14.75)	***P*** **< .01**
MDS-UPDRS IV	8 (4)	6 (5)	***P*** **< .001**	14 (5)	4 (2.5)	***P*** **< .001**
H-Y scale	3 (1)	2 (1)	*P* = .39	3 (1)	2 (0.25)	***P*** **= .03**
LEDD	1000 (550.5)	656 (629)	***P*** **= .0001**	1000 (247.125)	919 (328.125)	***P*** **< .01**

Significant differences at *P* < .05 are reported in bold

Patients who underwent GPi-DBS showed a significant improvement in motor experiences of daily living (MDS-UPDRS II: median 15, IQR 10 to median 10, IQR 8.5, *P* = .005) and motor symptoms severity, particularly when tested OFF state (MDS-UPDRS III, OFF: median 39, IQR 20.5 to median 25.5, IQR 14.75, *P* = .0003; MDS-UPDRS III, ON: median 17, IQR 19.5 to median 12, IQR 14, *P* = 0.07). These patients also showed a significant amelioration in motor complications (MDS-UPDRS IV: median 14, IQR 5 to median 4, IQR 2.5, *P* = .0007) and reduction in total LEDD (median 1000, IQR 247.125 to median 919, IQR 328.125, *P* = .005). Lastly, GPi-DBS reduced H-Y at the short-term evaluation (median 3, IQR 1 to median 2, IQR 0.25, *P* = .03), while no difference was found in the MDS-UPDRS I score.

### Long-term efficacy evaluation

The long-term evaluation was performed in a subset of 11 patients with STN-DBS and 10 with GPi-DBS. The median (IQR) scores at the clinical assessment in the whole cohort are summarized in Table 4.

**Table 4:** For each target, the table reports median value for each endpoint item at baseline and long-term evaluation

	STN			GPi		
	Pre DBS Median (IQR)	Long term Median (IQR)	*P* value	Pre DBS Median (IQR)	Long term Median (IQR)	*P* value
MDS-UPDRS I	10 (5)	13 (4)	*P* = .13	11.5 (5.75)	9 (6.5)	***P*** **= .04**
MDS-UPDRS II	17 (10)	15 (8)	*P* = .86	17 (9.5)	10.5 (6.75)	***P*** **= .02**
MDS-UPDRS III ON	22 (19)	12 (12.5)	***P*** **= .04**	13 (14.75)	11.5 (11.25)	*P* = .70
MDS-UPDRS III OFF	35.5 (7.75)	17.5 (10.5)	***P*** **= .03**	39 (19.5)	20.5 (16.25)	***P*** **= .05**
MDS-UPDRS IV	10 (4)	6 (7)	***P*** **= .02**	14.5 (1.75)	5.5 (3.5)	***P*** **= .005**
H-Y Scale	3 (0.5)	2 (1)		2.5 (1)	2 (1.5)	*P* = .11
LEDD	875 (262)	652.5 (306.25)	***P*** **= .04**	1000 (295.3)	936.5 (365.25)	*P* = .55

Significant differences at *P* < .05 are reported in bold

In the cohort of patients who underwent STN-DBS, clinical changes in the long-term matched those observed in the short-term evaluation. In particular, we found a significant improvement in motor symptoms severity (MDS-UPDRS III ON: median 22, IQR 19 to median 12, IQR 12.5, *P* = .04; MDS-UPDRS III OFF: median 35.5, IQR 7.75 to median 17.5, IQR 10.5, *P* < .05) and motor complications score (MDS-UPDRS IV: median 10, IQR 4 to median 6, IQR 7, *P* = .02), and a significant reduction in LEDD (median 875, IQR 262 to median 652.5, IQR 306.25, *P* = .0001). Again, there was no statistically significant difference in MDS-UPDRS I, MDS-UPDRS II, or H-Y score from baseline.

In the cohort of GPi-DBS patients, we found a significant improvement in the non-motor experiences of daily living compared to baseline (MDS-UPDRS I: median 11.5, IQR 5.75 vs. median 9, IQR 6.5, *P* = .04), which was not present at the short-term evaluation. In addition, at the long-term evaluation, patients showed amelioration in motor experiences of daily living (MDS-UPDRS II: median 17, IQR 9.5 vs. median 10.5, IQR 6.75, *P* = .02), motor symptoms severity OFF state (MDS-UPDRS III, OFF: median 39, IQR 19.5 vs. 20.5, IQR 16.25, *P* = 0.05) and motor complications scores (MDS-UPDRS IV: median 14.5, IQR 1.75 vs. median 5.5, IQR 3.5, *P* = .005). No difference from baseline was found in the MDS-UPDRS III (ON state), LEDD and H-Y scores.

### Comparison between STN-DBS and GPi-DBS clinical outcomes at short and long term

The comparison of changes in clinical outcomes from baseline to both short-term and long-term time points between the two targets is shown in Table 5.

**Table 5:** For each target, the table reports the median absolute variation (value at follow-up − value at baseline) for each endpoint item between baseline and both short-term and long-term evaluation

	Short term changes			Long term changes		
	STN-DBS Median (IQR)	GPi-DBS Median (IQR)	*P* value	STN-DBS Median (IQR)	GPi-DBS Median (IQR)	*P* value
MDS-UPDRS I	−2 (6)	−1 (7.5)	*P* = .93	+4 (8)	−4 (7)	***P*** **= .02**
MDS-UPDRS II	−1 (5)	−7 (7)	***P*** **= .03**	+3 (13)	−9 (7.75)	***P*** **= .02**
MDS-UPDRS III ON	−4 (12)	−5 (7)	*P* = .93	−5 (11)	−1 (11.75)	*P* = .23
MDS-UPDRS III OFF	−17 (13.75)	−16 (16.75)	*P* =.83	−17 (15.75)	−18 (18.5)	*P* = .34
MDS-UPDRS IV	−4 (6)	−9 (3)	***P*** **< .001**	−4 (5.5)	−9.5 (3.5)	***P*** **= .01**
H-Y scale	0 (0)	0 (1)	*P* = .11	0 (1.5)	0 (0.75)	*P* = .43
LEDD	−300 (400)	−50 (311)	***P*** **= .02**	−250 (381.2)	−25.37 (326.12)	*P* = .24

Significant differences at *P* < .05 are reported in bold

At short-term follow-up, we found no difference in the magnitude of improvement in motor symptoms severity (MDS-UPDRS III ON state: *P* = .93; MDS-UPDRS III OFF state: *P* = .83), non-motor experiences of daily living (MDS-UPDRS I: *P* = .93), and Hoehn–Yahr scale (*P* = .11) between the two targets. However, GPi-DBS was superior to STN-DBS in ameliorating motor experiences of daily living, motor fluctuations and dyskinesia, as reflected by the greater decrease in MDS-UPDRS II (STN-DBS median variation −1, IQR 5 vs. GPi-DBS median variation −7, IQR 7, *P* = .03) and MDS-UPDRS IV scores (STN-DBS median variation −4, IQR 6 vs. GPi-DBS median variation −9, IQR 3, *P* < .001). On the other hand, STN-DBS was associated with a greater reduction of LEDD (STN-DBS median variation −300, IQR 400 vs. GPi-DBS median variation −50, IQR 311, *P* = .02).

At the long-term follow-up, we found comparable changes in motor symptoms severity (MDS-UPDRS III ON state: *P* = .23; MDS-UPDRS III OFF state: *P* = .34), Hoehn–Yahr scale (*P* = .43) and LEDD (*P* = .24) between GPi-DBS and STN-DBS. Interestingly, GPi-DBS outperformed STN-DBS in improving motor and non-motor experiences of daily living (MDS-UPDRS I: STN-DBS median variation +4, IQR 8 vs. GPi-DBS median variation −4, IQR 7, *P* = .02; MDS-UPDRS II: STN-DBS median variation +3, IQR 13 vs. GPi-DBS median variation −9, IQR 7.75, *P* = .02) and in attenuating motor complications (MDS-UPDRS IV: STN-DBS median variation −4, IQR 5.5 vs. GPi-DBS median variation −9.5, IQR 3.5, *P* = .01).

### Safety evaluation

In the whole sample of 48 patients, we recorded 5 serious adverse events (10.4%), all occurring in PD patients with STN-DBS. Particularly, 2 patients experienced implant infections during the first year after surgery leading to the explant of devices, and 1 patient had an ischemic stroke during the implant procedure. Also, 2 patients committed suicide during the first year after surgery (one of them had a classic N370S mutation in the GBA gene, and the other had a single variant of uncertain significance in the NPC2 gene). In contrast, no adverse events occurred in patients who underwent GPi-DBS.

## Discussion and conclusions

In this retrospective study, we aimed at evaluating the efficacy and safety of DBS in our multicenter cohort of advanced PD patients and comparing short- and long-term clinical outcomes between patients treated with GPi-DBS and STN-DBS. We found that STN-DBS improved motor symptoms and complications and decreased LEDD in both the short and long term after surgery. GPi-DBS ameliorated motor dysfunctions, particularly in the OFF condition, and had a positive impact on motor complications and motor experiences of daily living. When comparing the two targets, we found no difference in motor symptom improvement. However, GPi-DBS was more effective in reducing motor complications and motor experiences of daily living at both time points than STN-DBS, which instead produced a greater decrease in LEDD.

The first result of our study concerns the clinical outcomes of DBS at the short-term follow-up. From a motor standpoint, both STN-DBS and GPi-DBS patients experienced a significant improvement in the MDS-UPDRS III score. Importantly, the direct comparison of the absolute changes from baseline to the short-term follow-up in motor severity outcomes did not show any difference between the two targets. The lack of significant differences was confirmed when evaluating patients both ON and OFF dopaminergic therapy. This result is not surprising, as fully in line with previous studies which demonstrated a substantial equivalence between the two targets in the treatment of motor symptoms [3, 5, 6]. Again, in keeping with the available literature, we also found a reduction in LEDD after both STN-DBS and GPi-DBS, which was significantly greater in the STN-DBS cohort [3, 5, 6]. Regarding motor fluctuations and dopaminergic therapy complications, we confirmed previous evidence that both STN-DBS and GPi-DBS improve the MDS-UPDRS IV score [3, 5, 6]. However, in our study, the improvement was more prominent in the GPi-STN group. Taking into account the different effects of STN-DBS and GPi-DBS on LEDD reduction, the variation in the MDS-UPDRS IV score can be attributed to separate phenomena in the two groups: in the case of STN-DBS, the improvement in motor complications can be attributed to the dopaminergic therapy decrease, while for GPi-DBS an intrinsic anti-dyskinetic effect of the stimulation can be hypothesized [19]. Although testing non-motor symptoms and quality of life was not among the study’s primary objectives, we detected a significant reduction in the MDS-UPDRS II scores only after GPi-DBS, suggesting a possible superiority of this target over STN on motor experiences of daily living.

Concerning the subgroup of patients with long-term follow-up data, similar to the short-term evaluation, motor symptoms severity improved after both STN-DBS (MDS-UPDRS III OFF and ON state) and GPi-DBS (MDS-UPDRS III OFF state), with no difference between the two interventions at the direct comparison analysis. In contrast, only STN-DBS patients experienced a significant long-term decrease in dopaminergic therapy, confirming previous studies [3, 5, 6]. Both targets achieved long-term beneficial effects on motor complications (MDS-UPDRS IV), but the amount of effect was significantly larger in GPi-DBS patients. This finding confirms the well-known greater effectiveness of GPi-DBS in treating l-dopa-induced dyskinesia and provides a specific indication for choosing this target in highly dyskinetic patients [20, 21]. Finally, concerning non-motor symptoms and quality of life, we observed a significant long-term improvement only in GPi-DBS patients. Overall, the aforementioned long-term data suggest a substantial non-inferiority of GPi-DBS in the treatment of motor symptoms and its superiority over STN-DBS in ameliorating motor fluctuations and dyskinesia and the experiences of daily living. We speculate that the latter two beneficial effects of GPi-DBS may be related, i.e., significant improvements in motor fluctuations and complications may have a positive impact on motor experiences of daily living in patients.

Genetic testing performed on our cohort showed a high percentage of patients with causative or risk-increasing mutations for PD, which appear to be higher than in previous studies [10, 22]. This finding could depend on a selection bias, whereby patients with early onset PD are generally well suited to surgery but also have a higher probability of carrying a mutation. However, it could also be due to our center’s frequent use of genetic testing for PD patients. In our study, genetic profiling was not used to drive target selection. Unfortunately, the results of genetic testing were available only in a small proportion of patients at the time of surgery. Our data are also insufficient to perform an adequate stratification of patients’ outcomes in relation to their genetic status. Genetic characterization of patients enrolled for DBS will become very important in the future, especially in the light of recent evidence on prognostic implications for patients with genetically determined PD undergoing STN-DBS, like GBA-mutated patients [23].

Regarding serious adverse events (SAEs), all were recorded in STN-DBS patients. Although we cannot exclude that the absence of SAEs in GPi-DBS patients was due to the smaller sample size, the fact that these were restricted to patients who underwent STN-DBS requires a separate comment. According to the CAPSIT protocol [2], severely depressed and psychiatric patients were excluded from the screening as possible DBS candidates. Despite this, two patients with STN-DBS committed suicide during the first year of follow-up. Examining the similarities and differences between these two patients, both had a good motor response to DBS treatment; one with N370S GBA mutation had 18 years of disease and died 10 months after the implant, while the second one had 11 years of disease and died 3 months after surgery. Suicide attempts and suicides have been reported in DBS patients, but the rate was reported not to differ from those on the best medical treatment (BMT) [24, 25]. A recent literature review highlights a higher risk of suicide associated with STN-DBS compared to GPi-DBS and tries to identify the responsible mechanism, citing neuroanatomical circuit dysfunctions and aberrant immunological activation as possible causes [26]. Other studies have highlighted the role of STN-DBS in the onset of impulsivity and compulsivity [27]. Regarding other adverse events, device infections are reported in 3.5% of total DBS patients, consistent with the data reported in the literature [28, 29].

Our study has some relevant limitations to mention. First, the sample size was small, especially the GPi-DBS cohort and the subgroup of patients followed up in the long term. This aspect makes it difficult to generalize our results to other groups of patients. Another limitation relates to the retrospective nature of the study. Finally, data collection and analysis focused on the motor aspects of the disease and DBS complications. Specific evaluations on non-motor aspects of the disease are missing.

In conclusion, our findings suggest that STN-DBS and GPi-DBS are equivalent in terms of effectiveness on motor symptoms, but the latter target appears superior in improving motor fluctuations and complications and, more generally, patients’ quality of life. Notably, although obtained in a small sample, these results were also observed in the long-term follow-up. Considering the long duration of DBS implants [30, 31], these data suggest that GPi-DBS could be an equally valid option to STN-DBS to treat advanced PD.

## Data Availability

Data are available on request to corresponding author.

## Abbreviations

DBS: Deep brain stimulation
STN: Subthalamic nucleus
GPi: Globus pallidus internus
UPDRS: Unified Parkinson’s disease rating scale
